# Taliglucerase alfa: safety and efficacy across 6 clinical studies in adults and children with Gaucher disease

**DOI:** 10.1186/s13023-018-0776-8

**Published:** 2018-02-23

**Authors:** Ari Zimran, Michael Wajnrajch, Betina Hernandez, Gregory M. Pastores

**Affiliations:** 1 Gaucher Clinic, Shaare Zedek Medical Center, Hebrew University and Hadassah Medical School, 12 Bayit Street, P.O. Box 3235, 91031 Jerusalem, Israel; 2 Pfizer Inc, New York, NY USA; 3 University College Dublin and the National Centre for Inherited Metabolic Disorders, Mater Misericordiae University Hospital, Dublin, Ireland

**Keywords:** Anaemia, Enzyme replacement therapy, Gaucher disease, Imiglucerase, Hepatomegaly, Paediatrics, Splenomegaly, Taliglucerase alfa

## Abstract

Taliglucerase alfa is an enzyme replacement therapy (ERT) approved for treatment of adult and paediatric patients with Type 1 Gaucher disease (GD) in several countries and the first plant cell–expressed recombinant therapeutic protein approved by the US Food and Drug Administration for humans. Here, we review the findings across six key taliglucerase alfa clinical studies. A total of 33 treatment-naïve adult patients were randomized to taliglucerase alfa 30 U/kg or 60 U/kg in a 9-month, multicentre, randomized, double-blind, parallel-group, dose-comparison pivotal study, after which eligible patients continued into two consecutive extension studies; 17 treatment-naïve adult patients completed 5 total years of treatment with taliglucerase alfa. In the only ERT study focused on exclusively paediatric patients with GD, 11 treatment-naïve children were randomized to taliglucerase alfa 30 U/kg or 60 U/kg in a 12-month, multicentre, double-blind study; nine completed 3 total years of treatment in a dedicated paediatric extension study. The effect of switching patients from imiglucerase to taliglucerase alfa was also investigated in a separate 9-month study that included 26 adults and five children; 10 adults completed a total of 3 years and two children completed a total of 2.75 years of taliglucerase alfa treatment in the extension studies. All studies evaluated safety and spleen volume, liver volume, platelet count, haemoglobin concentration, and biomarkers as measures of efficacy. Detailed results from baseline through the end of these studies are presented. Taliglucerase alfa was well tolerated, and adverse events were generally mild/moderate in severity and transient. Treatment with taliglucerase alfa resulted in improvements (treatment-naïve patients) or stability (patients switched from imiglucerase) in visceral, haematologic, and biomarker parameters. Together, this comprehensive data set supports the treatment of adult and paediatric patients with GD who are naïve to ERT or who have previously been treated with imiglucerase.

## Background

Gaucher disease (GD) is a rare lysosomal storage disorder caused by autosomal recessive mutations in the gene encoding β-glucocerebrosidase, a lysosomal enzyme required for the degradation of the glycolipid, glucocerebroside [1]. The mutations cause deficiencies in β-glucocerebrosidase activity, resulting in lysosomal substrate accumulation of glucocerebroside within macrophages, which become engorged Gaucher cells and cause multisystemic damage in organs and tissues, including adverse effects on the spleen, liver, bone, platelets, and haemoglobin [1]. There are three major types of GD [2, 3]: Type 1 is the non-neuropathic and most prevalent form of the disease and can manifest at any age, from infancy to adulthood; Types 2 (acute) and 3 (sub–acute) are neuropathic forms.

For more than 2 decades, enzyme replacement therapy (ERT) has been the mainstay of treatment for patients with Type 1 GD [2, 4] and is highly effective in reversing the visceral and haematologic manifestations of the disease [4]. In the United States and many countries in the European Union, three ERTs have been approved for the treatment of Type 1 GD: taliglucerase alfa, velaglucerase alfa, and imiglucerase. All three ERTs are recombinant active forms of β-glucocerebrosidase and are administered by intravenous infusion [5–8]. Both velaglucerase alfa and imiglucerase are produced in mammalian cell-based expression systems that require glycosylation modifications during production to expose the appropriate mannose residues needed for efficient cellular uptake of the recombinant enzymes, adding cost and additional steps to the production processes [4, 6, 7, 9–11]. Mammalian cell-based expression systems have also been vulnerable to the risk of supply shortage, such as the viral contamination of a bioreactor that prompted the temporary suspension of imiglucerase manufacturing [12].

Taliglucerase alfa, the most recently approved ERT for Type 1 GD, was initially approved in 2012 by the US Food and Drug Administration for the treatment of Type 1 GD in adults [8]. The US approval of taliglucerase alfa in adults was based on a phase 1 study performed in healthy volunteers, directly followed by two phase 3 studies—one in naïve adults and a second in ERT-experienced adults and children with Type 1 GD; there was a waiver obviating the need for a phase 2 study.

Taliglucerase alfa was thus approved for use in both adult and paediatric populations with Type 1 GD in multiple countries including the United States and Canada, in adult patients with Type 1 GD in Panama, and for treatment of haematologic manifestations in paediatric patients with Type 3 GD in a number of countries, including Canada, Colombia, Ukraine, and Taiwan. Despite the drug’s positive risk-benefit assessment, the European Medicines Agency recommended against marketing authorization for taliglucerase alfa in 2012 because velaglucerase alfa had received orphan market exclusivity in the European Union for Type 1 GD [13].

Taliglucerase alfa is produced in carrot cells and is the first recombinant therapeutic protein produced in a plant cell-expression system to be approved for use in humans by the US Food and Drug Administration [14]. The plant-based platform for producing taliglucerase alfa uses scalable, disposable bioreactors and is free of mammalian components [11]. In addition, the production process for taliglucerase alfa does not require additional steps to create the glycan structures necessary for cellular uptake by Gaucher cells [9, 10]. The novel and unique features of the production process for taliglucerase alfa offer potential benefits regarding lack of mammalian contaminants (e.g., providing a natural firewall against mammalian infectious vectors), scalability, and cost benefits associated with disposable bioreactors and lack of need for additional steps to ensure correct glycosylation for efficient cellular uptake. The enzymatic activity of taliglucerase alfa and its uptake into macrophages have been shown to be comparable to those of imiglucerase, further supporting its place among available treatments for GD [9–11]. The purpose of this review is to provide an overview of the results from the phase 3 clinical studies of taliglucerase alfa in adults and children with GD.

### Overview of taliglucerase alfa phase 3 clinical studies

**Fig. 1:**
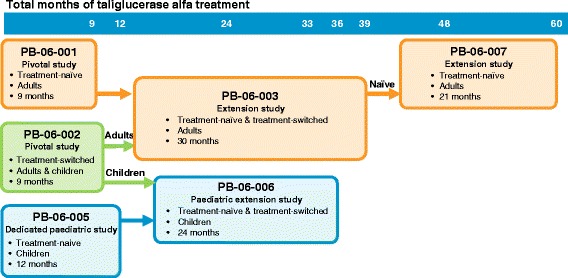
Taliglucerase alfa phase 3 clinical studies

Phase 3 clinical studies of taliglucerase alfa included adults ≥18 years of age and children 2 to < 18 years of age (combined: *N* = 73) who were either treatment-naïve (adults: *n* = 31; children: *n* = 11) or switched from imiglucerase to taliglucerase alfa (adults: *n* = 26; children: *n* = 5). The flow of these studies is summarized in Fig. 1, and patient disposition is summarized in Table 1. The detailed methodology and patient flow have been described previously [15–21]. A brief overview of the study designs, patient populations, treatments, and durations is provided in Table 2 [15–21].

**Table 1:** Patient disposition in the taliglucerase alfa clinical studies^a^

Patient population and study	Enrolled	Completed	Notes
Adult treatment-naive patients			
PB-06-001 [15]	33	29	• 1 patient withdrew consent before first dose • 3 patients discontinued
PB-06-003 [18]	26	23	• 3 patients not recruited to PB-06-003 • 3 patients discontinued
PB-06-007 [21]	19	17	• 4 patients not recruited into PB-06-007 • 2 patients discontinued
Adult treatment-switched patients			
PB-06-002 [16]	26	25	• 1 patient discontinued
PB-06-003 [19]	19	10	• 6 patients from PB-06-002 not recruited into PB-06-003, 5 of whom continued with compassionate use program • 1 patient non-compliant • 4 patients discontinued • 4 patients treated for 30–33 total months but transitioned to commercial use before study completion
Paediatric treatment-naïve patients			
PB-06-005 [17]	11	11	
PB-06-006 [20]	10	9	• 1 patient from PB-06-005 not recruited into PB-06-006 and continued with compassionate use program • 1 patient dropped out during PB-06-006
Paediatric treatment-switched patients			
PB-06-002 [16]	5	5	
PB-06-006 [20]	5	4	• 2 patients completed 27 months of treatment and continued with compassionate use program • 2 patients completed 33 months of treatment • 1 patient lost to follow-up

^a^For full patient disposition details, refer to the original studies. For some endpoints, data were not available for all patients; refer to the original studies for reasons

**Table 2:** Overview of the taliglucerase alfa clinical studies

Study^a^	Design	Patient population	*N* ^b^	Taliglucerase alfa treatment	Duration (Months)	Key efficacy findings	Key safety findings
PB-06-001 (NCT00376168) Zimran, 2011 [15]	• Multicentre • Double-blind • Randomized	• Adults ≥18 years • Treatment-naive	31	• Parallel dose • 30 U/kg or 60 U/kg	9	• Significant improvements in visceral and haematologic parameters and chitotriosidase activity	• TEAEs mild/moderate and transient • No SAEs
PB-06-002 (NCT00712348) Pastores, 2014 [16]	• Multicentre • Open-label • Switchover	• Adults (*n* = 26) and children (*n* = 5) > 2 years • Previously treated with imiglucerase	31	• Same dose as previously treated with imiglucerase (9–60 U/kg)	9	• Stability of visceral, haematologic, and biomarker parameters	• TEAEs mild/moderate and transient • No SAEs related to treatment • No discontinuations due to drug-related AEs
PB-06-003 (NCT0070593) Zimran, 2016 [18]; Pastores, 2016 [19]	• Multicentre • Double-blind and open-label • Extension	• Adults from PB-06-001 or PB-06-002 • Treatment-naïve (*n* = 26) or treatment-switched (*n* = 19)	45	• Same dose as in previous study	30	• Treatment-naïve: Visceral and biomarker parameters and platelets improved continuously; haemoglobin reached normal levels and stabilized • Treatment-switched: Continued stability or improvement	• TEAEs mild/moderate and transient • All SAEs not related to treatment
PB-06-005 (NCT01132690) Zimran, 2015 [17]	• Multicentre • Double-blind • Randomized	• Children 2–< 18 years • Treatment-naïve	11	• Parallel dose • 30 U/kg or 60 U/kg	12	• Clinically significant improvements in visceral, haematologic, and biomarker parameters • Trend toward improvement in exploratory parameters of growth and development • Treatment well tolerated	• Most AEs mild/moderate, transient, and not treatment-related • No deaths • No clinically meaningful laboratory abnormalities
PB-06-006 (NCT01411228) Zimran, 2016 [20]	• Multicentre • Double-blind and open-label • Extension	• Children from PB-06-005 (originally treatment-naïve; *N* = 10) • Children from PB-06-002 (treatment-switched; *N* = 5)	15	• Continued parallel doses at 30 U/kg or 60 U/kg • Continued at previous dose in PB-06-002	24	• Continued improvement (naïve) or stability (switched) in visceral, haematologic, and biomarker parameters • Continued improvement in exploratory parameters of growth and development • Treatment well tolerated	• All AEs mild/moderate • No deaths • No discontinuations due to AE • No clinically meaningful laboratory abnormalities
PB-06-007 (NCT01422187) Zimran, 2016 [21]	• Multicentre • Open-label • Extension	• Originally treatment-naïve adults from PB-06-003	19	• Continued parallel doses at 30 U/kg or 60 U/kg	21	• Visceral and biomarker parameters and platelets improved continuously; haemoglobin remained stable	• Most AEs mild/moderate and not treatment-related • Most laboratory values normal

^a^Study PB-06-004 was an open-label expanded access study; results will be reported separately

^b^Number of patients treated

AEs, adverse events; SAEs, serious adverse events; TEAEs, treatment-emergent adverse events

Primary and secondary efficacy end points included visceral (liver and spleen volume), haematologic (haemoglobin concentration, platelet count), and biomarker-related (chitotriosidase activity and/or chemokine C-C motif ligand 18 [CCL18 level]) parameters. Exploratory efficacy endpoints in the adult patients included changes in bone mineral density using dual-energy X-ray absorptiometry and a measurement of bone marrow fat fraction by quantitative chemical shift imaging [15, 21]. Paediatric exploratory efficacy endpoints included changes in height, weight, growth velocity, puberty, and bone age based on radiographs of the left hand and wrist, and occurrence of bone events (including bone crises as part of the analysis of adverse events [AEs]), quality of life using the 28-item Child Health Questionnaire parent-report questionnaire for patients 5 to 18 years of age, and sexual development as assessed by Tanner staging [17, 20]. Safety measurements in the clinical studies included AEs, immunogenicity (i.e., presence of anti-taliglucerase alfa IgG and IgE), assessment of neutralizing antibody activity using an in vitro assay and a cell-based assay, and assessment of infusion-associated reactions (i.e., hypersensitivity) [15–17, 19–21]. Additional safety evaluations included physical examination, vital signs, and clinical laboratory assessments, as well as electrocardiogram, echocardiogram, and pulmonary function tests.

### Efficacy

Key efficacy findings from the taliglucerase alfa phase 3 clinical studies are summarized in Table 2 [15–21], with additional efficacy results shown in Table 3 (treatment-naïve patients) [15, 17, 18, 20, 21] and Table 4 (treatment-switched patients) [16, 19, 20].

**Table 3:** Efficacy results for taliglucerase alfa in treatment-naïve patients

	Adults				Children		
	PB-06-001 [15] *N* = 32^a^		PB-06-003 [18] *N* = 26	PB-06-007 [21] *N* = 19	PB-06-005 [17] *N* = 11		PB-06-006 [20] *N* = 10
Parameter	Baseline	9 Months change from baseline^b^	36 Months	60 Months	Baseline	12 Months	36 Months
Spleen volume, MN							
30 U/kg	15 (*n* = 15)	−26.9% (*n* = 15)	8.2 (*n* = 12)	6.6 (*n* = 7)	22.2 (*n* = 6)	14.0 (*n* = 6)	9.0 (*n* = 4)
60 U/kg	17 (*n* = 16)	−38.0% (*n* = 16)	5.6 (*n* = 11)	3.2 (*n* = 7)	29.4 (*n* = 5)	12.9 (*n* = 5)	6.6 (*n* = 3)
Liver volume, MN							
30 U/kg	1.7 (*n* = 15)	−10.5% (*n* = 14)	1.3 (*n* = 12)	1.2 (*n* = 7)	1.8 (*n* = 6)	1.5 (*n* = 6)	1.3 (*n* = 4)
60 U/kg	1.6 (*n* = 16)	−11.1% (*n* = 15)	1.1 (*n* = 11)	1.0 (*n* = 7)	2.2 (*n* = 5)	1.7 (*n* = 5)	1.5 (*n* = 3)
Haemoglobin, mg/dL							
30 U/kg	12.2 (*n* = 15)	+ 1.6 (*n* = 14)	14.3 (*n* = 11)	14.1 (*n* = 7)	11.3 (*n* = 6)	12.7 (*n* = 6)	13.2 (*n* = 5)
60 U/kg	11.4 (*n* = 16)	+ 2.2 (*n* = 15)	14.0 (*n* = 11)	13.9 (*n* = 8)	10.6 (*n* = 5)	12.2 (*n* = 5)	12.7 (*n* = 4)
Platelet count, /mm^3^							
30 U/kg	75,320 (*n* = 15)	+ 11,427 (*n* = 15)	94,683 (*n* = 12)	104,986 (*n* = 7)	162,667 (*n* = 6)	208,167 (*n* = 6)	220,020 (*n* = 5)
60 U/kg	65,038 (*n* = 16)	+ 41,494 (*n* = 16)	147,727 (*n* = 11)	180,625 (*n* = 8)	99,600 (*n* = 5)	172,200 (*n* = 5)	243,750 (*n* = 4)
Chitotriosidase, percent change from baseline^c^							
30 U/kg	─	− 47% (*n* = 14)	− 71.5% (*n* = 12)	−83.1% (*n* = 7)	─	− 58.5% (*n* = 6)	−72.7 (*n* = 5)
60 U/kg	─	−58% (*n* = 15)	−82.2% (*n* = 10)	−93.4% (*n* = 7)	─	−66.1% (*n* = 4)	− 84.4 (*n* = 3)
CCL18, percent change from baseline^b^							
30 U/kg	─	NR^d^	− 58.1% (*n* = 12)	−66.7% (*n* = 7)	─	NR^d^	− 67.7% (*n* = 5)
60 U/kg	─	NR^d^	− 71.0% (*n* = 11)	−83.3% (*n* = 8)	─	NR^d^	− 73.2% (*n* = 4)

Values represent means

^a^32 patients were randomized; 31 received treatment

^b^Absolute values at 9 months were not reported; percent change from baseline was reported for organ volumes and absolute change from baseline was reported for haematological parameters

^c^To determine change from baseline for biomarkers, baseline values from entry into the original study (PB-06-001 for adults; PB-06-005 for children) were re-run at each time point due to high assay variability

^d^Values are available for individual patients in the publications cited

CCL18, chemokine (C-C) motif ligand 18; MN, multiples of normal where normal spleen volume is 2 mL/kg × body weight (kg) and normal liver volume is 25 mL/kg × body weight (kg); NR, not reported

**Table 4:** Efficacy results for taliglucerase alfa in treatment-switched patients

	Adults			Children		
	PB-06-002 [16] *N* = 26		PB-06-003 [19] *N* = 10	PB-06-002 [16] *N* = 5		PB-06-006 [20] *N* = 5
Parameter	Baseline	9 Months	33 Months, percent change from baseline^a^	Baseline	9 Months	33 Months, percent change from baseline^a^
Spleen volume, MN	5.5 (*n* = 20)	5.1 (*n* = 20)	−19.8% (*n* = 7)	4.1 (*n* = 5)	3.3 (*n* = 5)	−5.3% (*n* = 2)
Liver volume, MN	1.0 (*n* = 23)	0.9 (*n* = 23)	No change (*n* = 8)	1.3 (*n* = 5)	1.2 (*n* = 5)	− 8.8% (*n* = 2)
Haemoglobin, mg/dL	13.5 (*n* = 25)	Stable (*n* = 25)	−1.0% (*n* = 10)	13.5 (*n* = 5)	Stable (*n* = 5)	+ 3.3% (*n* = 2)
Platelet count, /mm^3^	160,447 (*n* = 25)	Stable (*n* = 25)	Nominal (*n* = 10)	164,587 (*n* = 5)	Stable (*n* = 5)	+ 2.3% (*n* = 2)
Chitotriosidase, percent change from baseline^b^	–	− 21.3% (*n* = 23)	−51.5% (*n* = 10)	–	− 29.7% (*n* = 5)	−97.1% (*n* = 2)
CCL18, percent change from baseline^b^	–	Decrease (*n* = 23)	−36.5% (*n* = 10)	–	Decrease (*n* = 5)	−10.8% (*n* = 2)

Values represent means

^a^Absolute values at 33 months not reported; percent change from baseline reported

^b^To determine change from baseline for biomarkers, baseline values from entry into the original study (PB-06-002) were re-run at each time point due to high assay variability

CCL18, chemokine (C-C) motif ligand 18; MN, multiples of normal where normal spleen volume is 2 mL/kg × body weight (kg) and normal liver volume is 25 mL/kg × body weight (kg)

#### Treatment-naïve adult patients with GD

At the end of the 9-month treatment period of pivotal Study PB-06-001 (treatment-naïve adult patients), all patients achieved the primary endpoint of reduction in spleen volume (30 U/kg: 26.9%; 60 U/kg: 38.0%; both *P* < 0.0001). In the 30 U/kg and 60 U/kg groups, respectively, significant reductions were also observed at 9 months versus baseline in liver volume (10.5%, *P* = 0.004, and 11.1%, *P* < 0.0001) and chitotriosidase activity (50% in both dose groups, *P* < 0.0001 and *P* = 0.0016), as well as a significant increase in haemoglobin concentration (1.6 g/dL, *P* = 0.001, and 2.2 g/dL, *P* < 0.0001). Platelet count increased significantly in the 60 U/kg group (41,494/mm^3^, *P* = 0.0031) and increased by 11,427/mm^3^ in the 30 U/kg group but did not achieve the prespecified alpha of 0.025 [15].

From baseline in original study PB-06-001 to the end of extension study PB-06-003 (up to 36 total months of treatment), taliglucerase alfa 30 U/kg and 60 U/kg, respectively, produced decreases in spleen volume (50.1% and 64.6%), liver volume (25.6% and 24.4%), chitotriosidase activity (71.5% and 82.2%), and CCL18 concentration (58.1% and 71.0%), and increases in haemoglobin concentration (16.0% and 35.8%) and platelet count (45.7% and 114.0%) [20]. At the end of adult extension study PB-06-007 (60 total months of treatment), the 30 U/kg, 60 U/kg, and dose-adjusted groups, respectively, demonstrated reductions in spleen volume (56.7%, 57.9%, and 61.0%), liver volume (32.5%, 23.3%, and 30.4%), chitotriosidase activity (83.1%, 93.4%, and 87.9%), and CCL18 concentration (66.7%, 83.3%, and 78.9%), and increases in haemoglobin concentration (2.1, 2.1, and 1.8 mg/dL) and platelet count (31,871, 106,800, and 34,000/mm^3^) versus PB-06-001 baseline [21]. In PB-06-007, lumbar spine fat fraction was assessed in a subset of patients, and clinically meaningful improvements occurred for this exploratory endpoint [21].

#### Treatment-switched adult patients with GD

Overall at the end of the 9-month treatment period in Study PB-06-002, disease parameters remained stable in patients who were previously treated with imiglucerase and switched to the same dose of taliglucerase alfa. Reductions were observed in spleen volume (7.6%), liver volume (3.5%), chitotriosidase activity (21.3%), and CCL18 concentration (value not reported) compared with baseline [16]. One adult patient had an increase in spleen volume, and one adult and one paediatric patient had increases in liver volume; these increases were not considered clinically meaningful and none of these patients experienced clinically relevant deteriorations in other efficacy parameters. One patient met the clinical protocol criteria for a sustained clinical deterioration in platelet count but improved to a platelet count of 170,000/mm^3^ by month 9 of treatment. Treatment-switched adult patients who had previously been in PB-06-002 generally demonstrated clinical stability for up to 36 total months in extension study PB-06-003, as evidenced by unchanged haemoglobin concentration, platelet count, and liver volume as well as reductions in spleen volume (19.8%), chitotriosidase activity (51.5%), and CCL18 concentration (36.5%) compared with baseline in original study PB-06-001 [19].

#### Treatment-naïve paediatric patients with GD

At the end of the 12-month study of treatment-naïve paediatric patients (PB-06-005), median percent changes (increases) in haemoglobin concentration (primary endpoint) were 12.2% for 30 U/kg and 14.2% for 60 U/kg (mean percent increases were 13.8% and 15.8%, respectively) [17]. From baseline to month 12, improvements were observed with decreases in absolute spleen volume in the 30 U/kg and 60 U/kg groups, respectively, of 28.6% and 41.1% and liver volume of 6.3% and 14.0%, as well as increases in platelet count of 30.9% and 73.7%, and decreases in chitotriosidase activity of 58.5% and 66.1% [17]. Analyses of exploratory endpoints for growth and development also trended toward improvement [17].

By the end of the paediatric extension study (PB-06-006), the patients who were previously in PB-06-005 (treatment-naïve paediatric patients) had received up to 36 total months of treatment. Patients in the 30 U/kg and 60 U/kg groups, respectively, achieved reductions in mean spleen volume (18.6 multiples of normal [MN] and 26.0 MN), liver volume (0.8 MN and 0.9 MN), and chitotriosidase activity (72.7% and 84.4%), and increases in haemoglobin concentrations (2.0 g/dL and 2.3 g/dL) and platelet counts (38,200/mm^3^ and 138,250/mm^3^) compared with baseline. Improvement in chitotriosidase and CCL18 continued through 36 total months of treatment, with most improvement noted by month 12 [20]. In treatment-naïve paediatric patients enrolled in PB-06-006, height increased by 12.4% (30 U/kg) and 19.2% (60 U/kg). Height velocity was 5.5 cm/year (30 U/kg) and 6.7 cm/year (60 U/kg). Weight increased by 39.8% (30 U/kg) and 35.0% (60 U/kg). Overall, eight of the 10 treatment-naïve paediatric patients had no change in pubertal status, as assessed by Tanner staging, while one patient progressed from Tanner stage 1 at baseline to stage 3 by the end of PB-06-006, and another patient advanced from Tanner stage 3 to stage 4 by 18 months. Over the 36-month trial period, bone age advanced by 3.6 years (30 U/kg) and 4.6 years (60 U/kg). In quality-of-life assessments, the number of parents/caregivers who rated their children’s global health as “very good” was greater at 36 months than at baseline [20].

#### Treatment-switched paediatric patients with GD

At the end of the 9-month treatment period of PB-06-002, all five children in the study remained clinically stable after being switched from imiglucerase to the same dose of taliglucerase alfa. Haemoglobin concentration and platelet concentration were unchanged, and reductions were observed in spleen volume (6.6%), liver volume (baseline: 1.3 MN; month 9: 1.2 MN), and chitotriosidase activity (29.7%) compared with baseline [16]. Treatment-switched paediatric patients from PB-06-002 who continued taliglucerase treatment in PB-06-006 maintained clinical stability. From baseline to up to 33 months, values remained stable or improved for spleen volume, haemoglobin concentration, platelet count, liver volume, chitotriosidase activity, and CCL18 concentration [20]. Exploratory growth and developmental endpoints of height and weight increased by 5.0% and 18.2%, respectively, and height velocity increased by 2.5 cm/year in treatment-switched paediatric patients [20]. Bone age increased by 2.3 years. Quality of life was not assessed in treatment-switched paediatric patients [20].

### Safety and immunogenicity

#### Treatment-naïve and treatment-switched adult patients

In PB-06-001 (treatment-naïve adult patients), no serious AEs were observed. The most frequently experienced treatment-related AEs were headache and pruritus. Two patients discontinued from the study due to a hypersensitivity reaction, and two patients developed anti-taliglucerase alfa antibodies that were determined to be non-neutralizing [15]. The most common AEs in extension study PB-06-003 (treatment-naïve adult patients) were arthralgia, headache, upper respiratory tract infection, pain in extremity, nasopharyngitis, and hypertension. Most (98.5%) AEs and all treatment-related AEs were mild or moderate in severity and transient in nature. Thirteen patients were found to have anti-taliglucerase alfa antibodies on at least one post-baseline visit; two of these patients were found to have neutralizing antibodies, but efficacy did not appear to be impaired by the development of neutralizing antibodies [18]. In Study PB-06-003 [18], the numbers of patients reported to develop anti-taliglucerase alfa IgG antibodies were higher than those reported in previous, shorter term studies [8, 15]. This was likely attributable to increased assay sensitivity due to assay modifications that resulted in differences in antibody sample positivity reporting. The assay modifications were made to establish statistically based cut-point definitions consistent with industry practices.

In extension study PB-06-007 (treatment-naïve adult patients), taliglucerase alfa was well tolerated. The most common AEs were nasopharyngitis and arthralgia. None of the AEs were related to treatment or led to study withdrawal, and 97.3% of the AEs were mild or moderate in severity. One patient who experienced hypersensitivity during PB-06-003 did not receive pre-medication and did not have a recurrence during PB-06-007. Eight patients had previously tested positive for anti-taliglucerase alfa antibodies before entry into PB-06-007. Five of those patients had also tested positive in PB-06-007, but did not discontinue and had no new treatment-related AEs. Three of the five patients tested positive for neutralizing activity using an in vitro assay [21].

In PB-06-002 (treatment-switched adult patients), the most frequent AEs reported in the adult patients were infusion-related reaction and nasopharyngitis. None of the severe or serious AEs were considered related to treatment. No discontinuations were due to a treatment-related AE, and all treatment-related AEs were mild or moderate in severity and transient in nature. Three patients tested positive for anti-taliglucerase IgG antibodies at some point during the study, including screening. Two of the patients were negative for neutralizing antibodies in the in vitro and cell-based assays, and neither experienced any treatment-related AEs. The third patient tested positive only in the in vitro assay [16]. In extension study PB-06-003 (treatment-switched adult patients), the most common AEs were nasopharyngitis, pyrexia, arthralgia, diarrhoea, vomiting, upper respiratory tract infection, cough, and musculoskeletal pain. Most AEs and all treatment-related AEs were mild or moderate in severity and transient in nature. Among the treatment-switched patients in PB-06-003, a total of four tested positive for anti-taliglucerase alfa IgG antibodies, including one patient who had evidence of neutralizing activity in an in vitro assay but who had a negative cell-based assay [19].

#### Treatment-naïve and treatment-switched paediatric patients

In PB-06-005 (treatment-naïve paediatric patients), most AEs were mild or moderate in severity, transient in nature, and not related to treatment. None of the AEs led to study withdrawal. One serious treatment-related AE (gastroenteritis requiring hospitalization for rehydration) occurred in a patient receiving taliglucerase alfa 60 U/kg during the first infusion visit; the incidence resolved after 1 day. None of the patients were diagnosed with a GD-related bone crisis during the study or experienced treatment-related bone pain. Three patients were positive for anti-taliglucerase alfa antibodies; all three patients had low titres, tested negative for neutralizing antibodies, continued to improve in GD parameters during the study with no apparent effect on efficacy and safety, and completed the study [17].

All AEs in PB-06-002 (treatment-switched paediatric patients) were considered mild or moderate in severity and unrelated to treatment. No severe AEs were reported in paediatric patients, and no children discontinued the study due to a drug-related AE. Anti-taliglucerase alfa antibodies were detected in two paediatric patients before treatment began and early on thereafter but not at later time points; both tested negative for the presence of neutralizing antibodies in the in vitro assay [16].

In extension study PB-06-006, which encompassed treatment-naïve paediatric patients from PB-06-005 and treatment-switched paediatric patients from PB-06-002, all AEs were mild or moderate in severity and none led to change in taliglucerase alfa dose or study discontinuation. The most common AEs were cough, headache, upper respiratory tract infection, abdominal pain, Dengue fever, diarrhoea, lymphedema, nasopharyngitis, and extremity pain. One AE was possibly related to treatment (mild Grade 1 infusion site pain in a treatment-naïve patient), but it resolved on the same day and was considered non-serious. One serious AE (Grade 2 Dengue fever) was reported in a treatment-naïve paediatric patient; the child was hospitalized and the event resolved within 5 weeks. No bone crises occurred during the study. One treatment-naïve paediatric patient who tested positive for anti-taliglucerase alfa IgG antibodies in PB-06-005 remained antibody-positive through PB-06-006 and tested positive for neutralizing antibodies based on the in vitro enzymatic activity assay; however, the child continued to show improvements in spleen and liver volumes, haemoglobin levels, platelet counts, chitotriosidase activity, and CCL18 levels through the 36 total months of taliglucerase alfa treatment. All paediatric patients in PB-06-006 who switched from imiglucerase tested negative for anti-taliglucerase alfa IgG antibodies during the extension study [20].

### Pharmacokinetics

The characterization of taliglucerase alfa pharmacokinetics (PK) was based on samples from 26 of the 31 patients in the pivotal PB-06-001 study (treatment-naïve adult patients) and 10 of the 15 patients in PB-06-006 (treatment-naïve and treatment-switched paediatric patients) [22]. Single-dose PK in adults were based on day 1 serial blood samples and multiple-dose PK were based on week 38 samples [22]. Multiple-dose PK in paediatric patients were based on samples collected after 10 to 27 months of treatment.

In adults and children, the 60 U/kg dose resulted in higher exposure (as measured by maximum plasma concentration, area under the plasma concentration-versus-time curve from time zero to the last measured concentration, and area under the plasma concentration-versus-time curve from time zero to infinity) than the 30 U/kg dose [22]. Within the adult and paediatric patient populations, the two dose groups were similar in mean time of maximum plasma concentration and elimination half-life values for taliglucerase alfa [22]. No tendency for accumulation or change in taliglucerase alfa PK was observed after repeated infusion with either dose over 38 weeks in adult patients [22]. Furthermore, dose-normalized exposure was comparable between adult and paediatric patients and showed dose proportionality in paediatric patients [22].

## Discussion

Treatment with taliglucerase alfa across six phase 3 clinical studies in treatment-naïve or treatment-switched adult and paediatric patients has been shown to result in clinically and statistically significant improvements in the major clinical features of Type I GD. Across these studies, taliglucerase alfa was well tolerated. Long-term improvements were observed in major GD disease parameters and biomarkers in treatment-naïve adult patients at dose levels of 30 U/kg and 60 U/kg, with a favourable tolerability profile and no new safety concerns over a 5-year treatment period [21]. Adult patients who were switched from imiglucerase demonstrated disease stability or improvements after a total of up to 3 years of treatment with taliglucerase alfa [19]. In treatment-naïve paediatric patients who received up to 3 years of treatment with taliglucerase alfa, continuous improvements were observed in visceral and haematologic parameters and biomarkers [20]. Paediatric patients who had switched from imiglucerase maintained clinical stability in these parameters during 33 months of treatment [20]. Growth inhibition and pubertal delay have been observed in children with GD [23–28], and exploratory analyses of growth and development in paediatric patients in the phase 3 taliglucerase alfa studies trended toward improvement in height and weight, progression of pubertal status, and absence of bone crises [20].

In the pivotal study of taliglucerase alfa in treatment-naïve adults with Type 1 GD (PB-06-001), the primary endpoint was reduction in spleen volume after 9 months of treatment based on magnetic resonance imaging (MRI) using validated automatic segmentation software in conjunction with a standardized MRI acquisition protocol [15, 29]. This primary endpoint was selected in part because changes in spleen volume have been shown to more closely reflect responsiveness to Gaucher-specific therapies than do changes in haematologic parameters. While haematologic parameters are also generally responsive to specific therapy, haemoglobin and platelet counts may be affected by conditions other than GD that may or may not be detected at screening. In addition, while many untreated patients with GD have anaemia, almost all untreated and symptomatic patients with GD have splenomegaly [30, 31]. In paediatric studies, use of MRI-based organ volume assessment may not be a first-choice approach because of potential concerns, such as inability to remain still and/or need for sedation during the MRI scan.

The formation of anti-drug antibodies is commonly observed with recombinant therapeutic proteins [11] and was observed in clinical studies of taliglucerase alfa but generally did not appear to be associated with the occurrence of AEs or a negative impact on efficacy. The clinical implications of seropositivity for anti-drug antibodies in the absence of AEs are unclear. In addition, comparing the incidence of antibodies to taliglucerase alfa with that of antibodies to other ERT products for GD or other recombinant therapeutic proteins should be approached with caution, as immunogenicity assay results can be highly dependent upon or influenced by extrinsic and intrinsic factors such as the sensitivity and specificity of the assay, assay methodology (including sample handling as well as timing of sample collection), concomitant medication, and underlying disease; hence, comparisons between studies and products may be misleading [8].

As with other studies of rare diseases, the taliglucerase alfa clinical studies in treatment-naïve patients with GD were limited by low patient numbers.

## Conclusions

Treatment with taliglucerase alfa resulted in long-term improvements (treatment-naïve patients) or stability (patients switched from imiglucerase) in visceral, haematologic, and biomarker parameters. Taliglucerase alfa is the only ERT for GD that was tested prospectively in paediatric-specific studies. Taliglucerase alfa was well tolerated, and AEs were generally mild or moderate in severity and transient in nature. Taken together, the comprehensive data set supports treatment with taliglucerase alfa in adult and paediatric patients with GD who are naïve to ERT or who have previously been treated with imiglucerase.

## Abbreviations

AEs: adverse events
CCL18: chemokine C-C motif ligand 18
ERT: enzyme replacement therapy
GD: Gaucher disease
MN: multiples of normal
MRI: magnetic resonance imaging
PK: pharmacokinetics
